# Diet, Exercise, Lifestyle, and Mental Distress among Young and Mature Men and Women: A Repeated Cross-Sectional Study

**DOI:** 10.3390/nu13010024

**Published:** 2020-12-23

**Authors:** Lina Begdache, Saloumeh Sadeghzadeh, Gia Derose, Cassandra Abrams

**Affiliations:** 1 Health and Wellness Studies Department, Binghamton University, Binghamton, NY 13902, USA; 2 School of Management, Binghamton University, Binghamton, NY 13902, USA; ssadeghz@binghamton.edu; 3 Department of Biological Sciences, Binghamton University, Binghamton, NY 13902, USA; gderose1@binghamton.edu; 4 School of Medicine, Stony Brook University, Stony Brook, NY 11794, USA; cassandra.abrams@stonybrook.edu

**Keywords:** young adults, mature adults, biological sex, mental distress, mental wellbeing, personalization of diet, brain maturity

## Abstract

Customization of mental health therapies needs to consider the differences in degree of brain maturity between young (18–29 years) and mature (30 years or older) adults as well as brain morphology among men and women. The aim of this study was to identify the significant dietary and lifestyle contributors to mental distress in these sub-populations. Independent repeated cross-sectional sampling was performed for over a 5-year period (2014–2019) to collect data from different populations at different time-points and seasons. A backward stepwise regression analysis was used on 2628 records. Mental distress in young women was associated with high consumption of caffeine and fast-food, and it was negatively correlated with moderate-high levels of exercise as well as frequent breakfast consumption. Mature women shared several common factors with young women; however, high fruit consumption was negatively associated with mental distress. For young men, high exercise, moderate consumption of dairy, and moderate-high intake of meat were negatively associated with mental distress. In addition, high fast-food and caffeine consumption were positively associated with mental distress in young men. For mature men, strong negative associations between higher education, moderate intake of nuts and mental distress surfaced. Our results support the need to customize dietary and lifestyle recommendations to improve mental wellbeing.

## 1. Introduction

The rising prevalence and awareness of mental distress, namely anxiety and depression, and their associated health care costs necessitate a change in disease management [1]. Mental distress negatively impacts every single aspect of life. It affects physical health [2], emotional eating [3], sleep [4], quality of life [5] and may lead to maladaptive coping mechanisms that are associated with alcohol and drug abuse [6]. To improve outcome, there is a need to move away from the one-size fits all approach and personalize mental health prevention and treatment strategies. The most common approaches employed include the use of antidepressants in combination with psychological counseling. One of the major modifiable risk factors for mental distress is the diet [7,8,9,10]. In fact, anxiety, depression, and other neuropsychiatric disorders are often associated with poor-quality diets [11,12,13]. Since the brain is a dynamic organ, a threshold of certain nutrients are necessary to support its function [14,15]. Nevertheless, there is a need to consider a couple of dimensions when personalizing dietary intake to support mental health. There is a difference in the prevalence of mental distress among young adults (18–29 years old) and their older counterparts (30 years and older) as well as between biological sexes [16,17]. In fact, young adults and women are at a higher risk of mental distress [17,18]. According to a recent report by the Centers for Disease Control and Prevention (CDC), U.S. young adults (age 18–29 years) exhibited the highest symptoms of mental distress (21.0%), in 2019, compared to all age groups. The same source reported that, during the same year, 21.8% of U.S women experienced symptoms of depression compared to men (15.0%) [19]. These discrepancies are due to the incomplete development of the prefrontal cortex (PFC) in young adults (YAs) [20] and the differential brain connectivity and cortical volume between men and women [21], respectively.

The first dimension to consider is the age bracket that accounts for the level of brain maturity. Human brain development continues well into the mid-to-late 20s [16]. Specifically, PFC is the last component of the brain to develop. PFC regulates several executive functions including feelings and rationalization of thoughts [22]. Although many environmental factors affect PFC maturity, the quality of the diet appears to have an impact [23]. Therefore, YAs who consume a poor-quality diet and experience nutritional deficiencies may suffer from a higher degree of mental distress.

The second dimension to consider is the biological sex, as brain morphology and connectivity is different between men and women [21,24,25]. Compared to men, women are more likely to suffer from longer episodes of mental distress with a higher chance for relapses [26,27]. Several imaging studies hint that differences in brain morphology is a potential cause. According to these reports, men have larger brain volume in subcortical regions after adjusting for age. The larger total brain volume is primarily in the grey matter (GM) of the subcortical regions [21], which is generally in information-processing areas. On the other hand, women have GM in the cortices and denser white matter (WM) between the frontal lobes, which consist of the regions that control impulses and emotions [28]. WM is comprised of myelinated axons that represent brain connectivity between different regions. Within WM, the myelin sheath is a fatty substance that coats axons to increase the speed of neuronal communication. It has fairly a high turnover rate, especially in the aging brain [29]. In essence, the male brain is wired for intra-hemispheric transmission while the female brain is optimized for inter-hemispheric communication [24]. This differential brain connectome is intended to enable perception and coordination in men, and to support analysis and intuition in women. Consequently, the dimorphic state of the brain may influence nutritional needs, behavioral traits as well as susceptibility to mood disorders.

Based on this line of evidence, distinct nutritional repertoires are allegedly recommended for age-groups and gender to optimize mental wellbeing in these sub-populations. To date, no research has explored these two premises. However, to improve the precision of the results, it is critical to assess other lifestyle factors that may influence mental health. Although there are several lifestyle factors that may impact mental status, to our knowledge, education, geographic locations, and seasonal change have not been studied in nutrition research in relation to mental distress. Therefore, the first aim of this study was to test the hypothesis that different frequencies of food groups and lifestyle factors are associated with mental wellbeing in young and mature men and women. The second aim was to identify the significant associations between dietary and lifestyle factors, including education, geographic locations, and seasons, with mental distress in these sub-populations [30,31,32]. Our findings could provide a proof-of-concept to support further research in the field of personalized therapy and mental health. They may also present a framework for developing dietary and lifestyle interventions as adjuncts to clinical and pharmacological therapies.

## 2. Materials and Methods

### 2.1. Study Design

This study was conducted according to the guidelines laid down in the Declaration of Helsinki, and all procedures involving research study participants were approved by the Institutional Review Board at Binghamton University. Independent repeated cross-sectional sampling was performed for over a 5-year period (2014–2019) to collect data from different populations at different times points and seasons. Dietary and nutrient consumption patterns were evaluated using the validated Food-Mood Questionnaire (FMQ).

### 2.2. Demographics

Adults of 18 years and older were invited to complete the questionnaire. FMQ was distributed to social and professional groups via several social media platforms. Participants consented to the study by agreeing to access the survey. There was no incentive or compensation provided for completing the questionnaire. Demographic questions included age, biological sex, dietary pattern followed, continent of residence, and highest education level attained. Geographical locations included North America, South America, Europe, Africa, Middle East/North Africa (MENA), Asia, and Australia/New Zealand. Due to a low number of responses from South America, Africa and Australia/New Zealand, these records were excluded from the analysis. Asia was considered in the northern hemisphere, as almost all of Asia is in the northern part. Date stamps along with geographical locations were used to identify the season. The following categorization was used: winter (December, January, February), spring (March, April, May), summer (June, July, August), and fall (September, October, November). Levels of education included less than high school, high school, two-to-four years of college degree, and graduate level degree (Master’s, Doctor in Philosophy and other professional degrees such as a Medical Degree).

### 2.3. Specifics of FMQ

FMQ is a 5-subscale item with an internal consistency, as reflected by Cronbach’s alpha values of greater than 0.70 for all sub-scales. FMQ is a reliable tool (Intra-class Correlation Coefficient of 0.619–0.884; *p*-value < 0.01; Confidence Interval (CI) 95%), which has an external validity as well [33]. FMQ was modeled after the National Health and Nutrition Examination Survey (NHANES) Food Frequency Questionnaire and gathers a concise dietary intake based on food group consumption and mental distress. Typically, food groups share common nutrients that have a direct effect on brain function [15]. FMQ evaluates weekly servings of nine different food groups: whole grain, fruits, vegetables, meat, beans and legumes, nuts, dairy, fish, and high glycemic index (HGI) foods. Answers ranged on a 6-level Likert scale (0 = none of the time to 6 = five times or more a week). Although there are daily recommendations set for nutrients, there is an increased appreciation for assessing dietary patterns rather than daily consumption of nutrients [34]. In fact, the USDA Healthy U.S.-Style Eating Pattern emphasizes the need to meet the weekly recommendations as the buildup of these nutrients confers health benefits [35]. Frequency of breakfast consumption and exercise (at least 20 min a day), use of multivitamins and fish oil supplements, and consumption of fast-food and caffeinated beverages (coffee, tea, soda, and energy drinks) were also assessed. Exercise frequency was based on the evidence from the literature stating that exercising for at least 20 min a day improves mental wellbeing [36,37,38]. FMQ includes the validated Kessler Psychological Distress Scale-6 (K-6), which uses a 5-level Likert scale for each question.

### 2.4. Sampling Techniques

Few recommendations from the literature were used to promote generalizability of the results. Kukull and Ganguli [39] advised to define the population of interest by establishing boundaries. For this research, mental distress in different age-groups and gender with diverse dietary patterns were selected as the boundaries. According to the authors “The population should include members with disease and members without disease.” Using the mental distress criteria provided by Prochaska et al. [40], we confirmed that some participants had no mental distress while others fell within a spectrum of mental distress. Additionally, Mullinex et al. [41] specifically discussed survey experiments by comparing results of convenience samples versus population samples and supported the use of “a multitude of samples for advancing scientific knowledge.” Therefore, a repeated cross-sectional design was applied, and a complete set of new respondents was constantly selected. Additionally, a combination of cluster and several non-probability sampling techniques were used to diversify participants, minimize bias and improve the external validity of the results. Data collection started in 2014 and continued intermittently until the end of 2019. Among the non-probability techniques, voluntary samplings and snowball sampling were employed. Cluster sampling was used to maximize the likelihood of a diverse population. It involved a careful selection of different social and professional groups that included international members. The aim was to reach out to heterogeneous populations cross-sectionally at different time points. The criteria selected for the social and professional groups are: (a) Groups should have more than 5000 members with no focus on a specific region. (b) Groups should be randomly selected if they perceptibly had no commonality of potential members, met the number of followers, and used the English language as the main language of communication. One advantage for using a web-survey is the absence of the interviewer’s influence. The fact that the survey was entirely anonymous may have supported candid responses. In fact, research on self-reported data describes that responses tend to be accurate when there is a great sense of privacy and lack of associated stigma [42].

### 2.5. Data Categorization

Data were categorized into four separate groups: Young women (age 18–29 years), Young men (age 18–29 years), Mature women (30 years or older), and Mature men (30 years or older) to assess dietary and lifestyle factors that associate with mental distress in these individual groups. Classification of the age-groups was based on brain maturity as suggested by Somerville [16]. Although early mature adults (30–50 years) and late mature adults (60 years or older) may exhibit differential prevalence of depression and anxiety due to brain changes, there is evidence that following a healthy diet and lifestyle may offset these structural alterations [10,43,44]. The dietary variables that were assessed include: whole grain, dairy, caffeine, fruits, nuts, HGI food (including rice and pasta), meat, vegetables, beans, fish, and fast-food. Frequency of breakfast consumption (Breakfast) and exercise (Exercise), as well as use of multivitamins (Multi-Vitamins) and fish oil (Fish Oil) supplements were included in the model. Additional variables considered included season (Spring, Summer, and Fall and Winter), continent of residence, and education level (Education). Dietary habits and exercise were classified as Low (0–1 time), Moderate (2–3 times), and High (4 times or more), based on recommendations set by the Dietary Guidelines for Americans (2015–2020) [45], and references such as ChooseMyPlate.gov and Office of Health Promotion and Disease Prevention for nutrients with no specific weekly recommendations. The reason for using three different categories was to reflect on the various relationships (i.e., linear versus U-shaped effect) with mental distress for several food groups. In addition, this categorization promotes accuracy by reducing the noise in data analysis. Mental distress was assessed using the total score of Kessler Psychological Distress Scale (K-6) questionnaire to account for the spectrum of mental distress as suggested by Furukawa et al. [46]. For each of the six categories (nervous, hopeless, restless, depressed, everything is an effort, and worthless), scores ranged between 0 and 4, where 0 characterized “None of the time” and 4 represented “All of the time.” In the regression-based models, individuals were classified into three different categories, as proposed by Krynen et al. [47], based on the total K-6 score: Low risk (0–4), Moderate risk (5–12), and High risk (13–24).

### 2.6. Statistical Analysis

Statistical relationships between variables were measured through regression-based models for each of the subgroups. For all models, a multivariate ordinal logistic regression was used to control several confounders in the dataset. To improve validity of the results and increase generalization of our models, a repeated 5-fold cross-validation was performed along with a backward stepwise variable selection. The best-fit model around different dependent and independent factors was selected by applying a backward stepwise variable selection based on the Akaike Information Criterion (AIC) obtained for all models [48]. Using the AIC criterion, an ordinal logistic regression that includes all the possible variables was performed to determine the model performance. Consequently, the variables were ranked based on their individual impact on mental status. At each iteration, the least important variables were removed, and the model was built on the remaining variables. The model with the best performance, based on the lowest AIC, was selected. Consequently, an ordinal logistic regression was performed to choose the best-fit model by examining the effect of each of the selected variables on mental status. A repeated 5-fold cross-validation was applied on the dataset to tune the parameters of the models. Data were randomly partitioned into five (nearly) equal subsets. Then, one of the subsets was chosen to serve as the validation set and the remaining four subsets were used to train the model. This process was repeated five times, i.e., until each subset was used exactly once as the validation set, and repeated the entire process 10 times, with 10 different random seeds to partition the training dataset. The data were examined closely for any duplicate responses. Missing data were handled by using imputation algorithms. Specifically, we used the k-Nearest Neighbor algorithm to predict the missing values by considering the rest of the available variables as significant contributors. Data suitability and sampling adequacy were confirmed by Bartlett’s test of sphericity (with significance level of 0.05). Data were analyzed in R, v3.5.0. (R Foundation for Statistical Computing, Vienna, Austria).

## 3. Results

A total of 2628 complete records from North America, Europe, MENA, and Asia were analyzed (Table 1). The breakdown of participants was as follows: 1147 young women, 628 mature women, 641 young men, and 207 mature men. The corresponding estimated margin of error with a 95% confidence interval is: 1.88%, 3.39%, 3.35% and 5.66%, respectively. Interestingly, young men and women exhibited significantly higher levels of mental distress compared to their mature counterparts. In addition, the spring season and living in the MENA region have a negative impact on the mental wellbeing of women and mature individuals, respectively.

### 3.1. Young Women

The relationship between mental distress and significant variables was assessed by performing a backward stepwise variable selection. The best-fit model for young women is as follows:

Mental status ~ Spring + Exercise + Breakfast + Caffeine + Fast-Food.

The results of the regression model for these selected variables are shown in Table 2. Based on the regression analysis, young women have a higher chance of mental distress during the spring season. High consumption of caffeine as well as moderate-to-high frequency of fast-food can negatively impact their mental health. Both moderate-to-high frequency of exercise and frequent breakfast consumption was significantly associated with mental wellbeing of young women.

### 3.2. Mature Women

A backward stepwise variable selection was repeated for mature women. The best-fit model of independent variables is as follows:

Mental Status ~ Spring + Asia + MENA + Exercise + Breakfast + Caffeine + Fruits + HGI Food + Vegetables + Beans.

Several factors that affected young women’s mental wellbeing, such as the spring season, caffeine, and fast-food consumption, resurfaced for mature women. However, unlike young women, only frequent exercise was associated with mental wellbeing. As for breakfast, moderate-to-high consumption was negatively associated with mental distress. New significant variables emerged for mature women. Living in Asia or in the MENA region, in comparison to living in North America, was associated with higher risk for mental distress. Moreover, high consumption of fruits was positively associated with mental wellbeing (Table 3).

### 3.3. Young Men

For young men, the best-fit model of independent variables chosen by the backward stepwise variable selection is as follows:

Mental Status ~ Summer + Asia + Exercise + Dairy + Caffeine + Meat + Vegetables + Beans + Fish + Fast-Food.

The results revealed that frequent exercise, moderate consumption of dairy, as well as moderate and high consumption of meat negatively was associated with mental distress. In addition, moderate-to-high fast-food and high caffeine consumption is positively associated with mental distress in young men (Table 4).

### 3.4. Mature Men

Following a backward stepwise variable selection, the best-fit model of independent variables for mature men is as follows:

Mental status ~ Asia + MENA + Education + Exercise + Whole Grain + Dairy + Caffeine+ Fruits + Nuts + HGI Food + Meat + Vegetables.

As with mature women, geographical locations and mental distress resurfaced for the mature men. Those who live in the MENA region have a higher chance of mental distress, in comparison to those who live in North America. Higher education and moderate consumption of nuts were negatively associated with mental distress in this category (Table 5).

## 4. Discussion

The purpose of this study was to test the hypothesis that different frequencies of food groups associate with mental wellbeing among young and mature men and women. Another aim was to identify the significant dietary and lifestyle factors that associate with mental distress in these sub-populations. To promote accuracy of the generated models, known confounding factors such as season, geographical locations, and educational attainment were assessed. Food groups, exercise frequency and other lifestyle factors were classified into low, moderate, and high, based on weekly recommendations. The current study is reporting several novel and significant findings. Our hypothesis that differential frequencies of food groups are associated with mental distress in young and mature men and women was confirmed. Interestingly, the spring season increases the chance of mental distress in young and mature women, and geographical locations impact the mental wellbeing of mature men and women. Moreover, mental distress is more prevalent in young adults and women (Table 6), which align with the CDC findings [19]. Our results confirmed our hypothesis that differential frequencies of dietary and lifestyle factors are associated with mental health in young and mature men and women.

### 4.1. Dietary and Lifestyle Approaches to Improve Mental Wellbeing among Young and Mature Women

Based on our results, the significant dietary and lifestyle approaches to improve mental wellbeing among young women include daily breakfast consumption, moderate-to-high exercise frequency, low caffeine intake and abstinence from fast-food. Conversely, the significant dietary and lifestyle approaches to improve mental wellbeing among mature women include daily exercise and breakfast consumption as well as high intake of fruits with limited caffeine ingestion. Additionally, a special attention to mental distress should be given to young and mature women during the spring season.

#### 4.1.1. Mental Distress and the Spring Season

According to our results, young and mature women are at a higher risk for mental distress in the spring. Although many environmental factors intertwine to impinge on mental health, the most significant causal factor reported is the daylight-saving time in the spring. This subtle change has been associated with a disordered circadian rhythm and sleep deprivation mostly in women [49]. A misalignment of the circadian rhythm and sleep disturbances are associated with higher risk of mental distress [50]. The circadian rhythm controls expression of a number of genes involved in several metabolic pathways [51]. This body clock is orchestrated by neural and endocrine mechanisms that stem from the master circadian pacemaker located in the hypothalamic suprachiasmatic nucleus (SCN) [52]. SCN is a multi-neuronal oscillator that works in concert to generate a lucid output rhythm and controls several bodily functions. Changes in daylight interfere with the phase-synchronization between individual neurons, which causes physiological and biochemical disruptions that impact mental health [53]. Inherently, women are more prone to disturbances in the circadian rhythm than men [54], which make them more susceptible to major depressive disorders with a seasonal pattern [55,56]. Timing of clock gene rhythms in the brain [57], diurnal body temperature regulation, production of melatonin and duration of sleep cycles tend to be shorter or occur at earlier stages in women [51]. Therefore, this sensitivity to alteration in daylight shifts several metabolic pathways that disturb neurotransmission [58]. Another theory explaining the risk of mental distress during springtime is the increase in allergic rhinitis (AR). AR promotes a large-scale inflammatory response by releasing several cytokines that induce neuroimmune reactions leading to psychiatric disorders [59]. In fact, high levels of chemokines were reported in patients with mental illnesses [60]. Mechanistically, cytokines activate the kynurenine pathway, which depletes the serotonin precursor, tryptophan, and produces neuroactive metabolites that disturb dopamine and glutamate neurotransmissions. This neuro-disruption impacts neurocircuits of several brain regions that control emotions such as the basal ganglia and anterior cingulate cortex [61]. Chronic activation of this innate immune response leads to depression and anxiety disorders in vulnerable individuals, such as females who produce serotonin at a slower rate compared to males [62].

#### 4.1.2. Mental Distress and Caffeine Metabolism

The hepatic metabolism of caffeine and steroid hormone competes for the same cytochrome P450 1A2 enzyme [63]. Therefore, the breakdown of caffeine is reduced in the presence of circulating estrogen, use of oral contraceptive, or hormone replacement therapy [64,65]. Consequently, an increase in estrogen levels slows down caffeine metabolism [66], which could double caffeine’s half-life [63]. Caffeine modulates the hypothalamic-pituitary-adrenocortical (HPA) axis and elevates glucocorticoid levels that activate the sympathetic nervous system [67]. Additionally, caffeine is an antagonist of the adenosine A_1_ and A_2A_ receptors, which are present in brain regions that process threat, fear, and anxiety [68,69,70]. Therefore, caffeine consumption increases risk of sleep problems, anxiety, and mood disorders [51,54]. This explains why caffeine intake in women is positively associated with shorter leukocyte telomeres, typical biomarkers of psychological stress and mental distress [71,72].

#### 4.1.3. Fruit Consumption and Mental Distress among Mature Women

Age is intrinsically coupled with altered immune functions that comprise the integrity of the blood–brain and the blood–cerebrospinal fluid barriers [73]. The function of these barriers is mainly to maintain homeostasis and confer neuroprotection. Therefore, seepage of potential neurotoxins into the brain results in inflammatory response activation, oxidative stress, and subsequent neuronal damage. Since women have higher levels of brain connectivity, they are more likely to be susceptible to faulty neurotransmission due to microlesions in the myelin sheath induced by inflammation and oxidative stress. Therefore, women may need higher levels of antioxidants, especially with declining estrogen levels [74,75]. These facts may explain our finding that fruit consumption is positively associated with mental wellbeing in mature women. In fact, supplementing with dietary antioxidants significantly reduced anxiety and depression scores of patients with mental health ailments [76]. In addition, low fruit consumption is associated with an increased risk of mental distress in adults 30 years or older [77]. Fruits contain a repertoire of potent antioxidant phytochemicals such as anthocyanin, flavonoids, carotenoids, and flavanols. Many of these biochemical compounds enhance brain function by elevating levels of brain-derived neurotrophic factor (BDNF) [78], which has anti-inflammatory and anti-apoptotic characteristics [79]. Additionally, BDNF promotes myelination, neuroplasticity, and neural repair [80], which are crucial for the aging brain.

#### 4.1.4. Exercise and Mental Wellbeing

The association between exercise and mental health is well established [36]. The finding from our study about the correlation between high exercise (only) and mental wellbeing among mature women is significant. Exercise promotes BDNF release, which is associated with improved brain function and mental wellbeing [81]. Interestingly, the promoter region of the BDNF gene comprises an estradiol-response-like element [82], which makes the degree of BDNF expression dependent on estrogen levels in women. Therefore, as estrogen levels decrease in mature women, BDNF levels dwindle down and induce changes in brain function. Several impaired cognitive functions in older women are associated with low BDNF levels [83]. However, an animal study suggested that the BDNF-induced adult hippocampal neurogenesis is more pronounced in females in response to stimuli [84], which could include physical exercise. In fact, Kurdi and Flora [85] reported that regular physical exercise boosted BDNF levels in peri-menopausal women, and the increase was higher in depressed women. This fact may support the higher need for mature women to exercise to promote their mental wellbeing. In accordance with these observations, our findings suggest that frequent exercise may compensate for the estrogen-decline-induced mental distress and boosts mental wellbeing in mature women.

### 4.2. Dietary and Lifestyle Approaches to Improve Mental Wellbeing among Young and Mature Men

Based on our findings, the significant dietary and lifestyle approaches to improve mental wellbeing of young men include frequent exercise, moderate dairy consumption, high meat intake, as well as low consumption of caffeine, and abstinence from fast-food. As with women, the association between high caffeine consumption and mental health in young men was expected due to the competition between caffeine and steroid hormone metabolism described earlier. Interestingly, there is an upward trend detected where higher meat consumption is associated with a greater mental wellbeing in young men (Table 4). This trend is consistent with findings from previous studies [77,86]. Additionally, the inverse link between exercise and mental distress was formerly described [38,77]; as well as the association between high fast-food consumption and mental distress in adolescents/young adults [77,87]. However, our current study is linking these significant variables to young men, as a specific sub-population to be at risk. Surprisingly, distinctly from young women, seasonal variations do not seem to impact young men’s mood. However, like young women, geographical locations do not influence the mental health of young men.

Again, as with mature women, geographical locations seem to significantly impact mood among mature men. In fact, the strongest contributor to mental distress is living in the MENA region, which is consistent with Baxter et al. [88] findings. According to the authors, anxiety among both genders is high in the MENA region mostly due to the ongoing conflicts. However, the strong positive factors that were associated with mental wellbeing among mature men are moderate intake of nuts and higher education. Interestingly, nuts and higher education support the intra-hemispheric arrangement of the male’s brain discussed earlier. Regular nut consumption strengthens brainwave frequencies associated with different cortical regions [89]. Higher education improves brain efficiency and may alleviate the impact of age on brain functional connectivity [90].

#### 4.2.1. Animal Proteins, Men’s Maturing Brain and Mental Wellbeing?

Our findings suggest that higher animal protein, namely meat, may be needed for the male maturing brain. However, evidence on the effect of animal protein on mood is controversial in the literature [86,91,92,93,94,95,96]. The mixed results potentially stem from the fact that several studies included both biological sexes and a wide-range of age-groups in their analysis. However, studies that distinctly categorized the sample population based on age-groups [77] or biological sexes [86] described an association between low meat consumption and mental distress. A large-scale study performed on 9668 young men, partners of pregnant women, reported that those who followed a vegetarian-style diet with high consumption of plant food scored higher on the depressive scale [86]. According to the authors, meat and mood may be following a U-shaped curve trend, i.e., while moderate consumption of meat improves mental health, very low and very high meat consumptions appear to be negatively associated with mental wellbeing [86]. Low meat or animal protein intake may increase risk of vitamin B_12_ and other nutrients deficiencies that disturb neural functions. Moreover, meat specifically is rich in bioavailable zinc and iron, which are essential cofactors for several biochemical pathways necessary for the maturing brain [97]. Therefore, subclinical deficiencies may alter brain homeostasis and induce mood changes. Although limiting animal protein intake provides several health benefits [98], poorly planned diets may have adverse health effects. Deficiencies in essential amino acids affect brain functionality by disrupting protein synthesis [99], which may reduce resilience to mental distress. On the other hand, a high consumption of animal protein may be part of the Western-dietary pattern that excludes fruits and vegetables. A high-protein intake with limited plant-based soluble fiber and polyphenols induce gastrointestinal dysbiosis, which has been linked to a decline in brain functions and mood [100]. Since men have a larger total cortical volume, regular consumption of animal products may be necessary to provide the essential building blocks for larger cortices.

The puzzling question that arises based on our results is why high meat consumption is important for the mental health of young men but not that of young women? The answer may lie in the kinetics of the intestinal metal transporters. Iron absorption from the gut is a highly regulated phenomenon. It involves several transport proteins, namely the divalent metal transporter 1 (DMT1), which carries other divalent metals such as zinc and copper [101]. As its name indicates, DMT1 transports the divalent iron, better known as ferrous iron or heme-iron, found in meat. During low levels of iron in females, potentially induced by regular menstruation, DMT1 expression increases to promote ferrous iron and other divalent iron absorption [102]. Clinical studies reported elevated levels of estrogen are associated with enhanced serum iron due to reduction in hepcidin synthesis, which improves serum iron level in menstruating females [103]. In addition, there is a positive association between iron and zinc status in premenopausal women [104], which supports the notion that iron and zinc absorption kinetics may be enhanced in young women.

#### 4.2.2. Men, Meat Consumption, High Exercise Frequency, Higher Education from an Evolutionary Point of View

To better understand the complex relationship between meat, exercise, higher education and mental health in men, looking into the human brain evolution may provide some insights. Meat consumption was prominent in early humans and is speculated to have contributed along with a nutrient dense-diet to the evolution of the human brain [105,106,107]. The latter involved growth of cortices and extensive neural wiring, which culminated in higher cognitive and behavioral aptitudes in modern humans [108].

Our ancestors exhibited gender-based physical and emotional responsibilities that have potentially dictated food preferences. Men were involved in intensive labor, which imposed a need for a larger muscle mass [109] and may have prompted an enhanced appetite for meat [110]. Meat supplies ample amounts of essential amino acids (AA), namely the branched chain AA, and creatine that promote muscle growth and power, respectively. Moreover, meat has several dietary components that support neurogenesis of the larger cortices identified in the contemporary male brain. In fact, vitamin B_12_, methionine, taurine, heme-iron and zinc work synergistically to preserve GM volume [111,112,113], which are associated with improved cognitive functions and emotional control [114,115,116]. In addition, vitamin B_12_ is an integral part of the one-carbon metabolism pathway that shaped the contemporary human brain through epigenetic modifications [117]. Accordingly, meat consumption in young men is potentially a physiological necessity that may have shaped the dimorphic brain. Although meat provides all the essential elements to support brain development and function, it is worth noting that brain function could still be optimized with a well-planned meatless diet. The careful planning of the diet is needed to avoid deficiencies in crucial elements needed for brain maturity and function.

Moreover, the intricate hunting tasks that were physically and mentally challenging for young men are comparable in intensity to the modern exercise, and allegedly supported the evolution of the human brain [118] through a continuous release of BDNF [119]. Although our results did not reveal that exercise is associated with mental wellbeing in mature men, higher education emerged as a positive contributor instead. In fact, higher education, which happens over several years, is associated with enhanced neuroplasticity, typically mediated by BDNF [120]. This notion was further supported by an in vitro study describing that overexpression of BDNF is coupled specifically with enhanced hippocampal neurogenesis [121], which is linked to improvement in mental wellbeing especially in men [109].

### 4.3. Strengths and Limitations of the Study

The strengths of the study include the heterogeneity of the population studied and the large sample size, which support generalizability of the results. In addition, several steps were undertaken to minimize bias. Our findings were generated using a robust analytical method, which strengthens the weight of the results. This study is adding to the literature by suggesting the need to customize approaches to promote mental wellbeing based on age-groups and biological sexes. It is also identifying the specific dietary and lifestyle factors that are strongly associated with mental health in these sub-populations. Nevertheless, the limitations of this study include its cross-sectional nature, nonrandom sampling and the smaller sample size for mature men. Although there is some evidence that men may not require a spectrum of nutrients to support mental wellbeing as women do, based on brain morphology [122], the current findings are presented with caution as the margin of error for this sub-population is larger than the rest of the sample. Increasing the sample size for this group is needed to confirm our findings. Nevertheless, our current analysis contains enough large sample sizes for the remaining three categories [123]. Moreover, the study does not take into consideration the variations in dietary patterns over the years that may have shaped their brain anatomy. In addition, there is no assessment of genetic variations, existing health conditions or environmental factors that may have shaped the psychology of the individuals.

## 5. Conclusions

Our findings revealed that common and differential frequencies of dietary and lifestyle factors are associated with mental wellbeing among young and mature men and women. Following a healthy dietary pattern and lifestyle is always recommended to improve health. As for mental health, no specific commendations within the established healthy recommendations have been proposed. Our results suggest that specific practices along with a consumption of a nutrient-dense diet are critical to promote mental wellbeing among young men and women.

Our results suggest that specific practices along with a consumption of a nutrient-dense diet are critical to promote mental wellbeing among young men and women. Although the notions of personalization or precision medicine refer to treatment centered around an individual’s genotype, mental health remedies based on gender and age groups may support a better outcome, as they take into consideration the level of brain maturity and differences in brain morphology. Therefore, to improve prognosis, a comprehensive approach needs to be implemented and a precision in dealing with the growing problem of mental distress is necessary.

## Figures and Tables

**Table 1:** Basic characteristics including number of participants categorized by age-group and sex.

Independent Variables	Age 18–29	Age 30 and Older	Total
*Biological Sex*			
Women	1147	628	1780
Men	641	207	848
*Geographical Location*			
Asia	109	68	177
North America	1319	423	1742
Middle east/North Africa (MENA)	247	271	518
Europe	113	76	189

**Table 2:** Regression analysis of mental distress and significant variables for young women.

Independent Variable	Coefficient	Standard Deviation	*t*-Value
Spring Season	0.533	0.148	3.595 *
Exercise (Moderate)	−0.402	0.153	−2.620 *
Exercise (High)	−0.653	0.167	−3.899 *
Breakfast (Moderate)	−0.425	0.221	−1.922
Breakfast (High)	−0.672	0.190	−3.532 *
Caffeine (Moderate)	−0.044	0.166	−0.262
Caffeine (High)	0.391	0.147	2.654 *
Fast Food (Moderate)	0.613	0.139	4.418 *
Fast Food (High)	1.029	0.217	4.733 *

* Statistically significant with α = 0.05.

**Table 3:** Regression analysis of mental distress and significant variables for mature women.

Independent Variable	Coefficient	Standard Deviation	*t*-Value
Spring Season	1.145	0.300	3.818 *
Asia	0.956	0.293	3.264 *
MENA **	0.884	0.185	4.782 *
Exercise (Moderate)	0.003	0.203	0.015
Exercise (High)	−0.668	0.220	−3.043 *
Breakfast (Moderate)	−0.690	0.313	−2.202 *
Breakfast (High)	−0.541	0.244	−2.219 *
Caffeine (Moderate)	0.346	0.252	1.372
Caffeine (High)	0.793	0.234	3.392 *
Fruits (Moderate)	−0.267	0.221	−1.205
Fruits (High)	−0.491	0.236	−2.078 *
HGI Food (Moderate)	0.109	0.201	0.542
HGI Food (High)	0.455	0.236	1.923
Vegetables (Moderate)	−0.164	0.324	−0.506
Vegetables (High)	−0.591	0.329	−1.796
Beans (Moderate)	−0.081	0.180	−0.449
Beans (High)	0.612	0.317	1.934

* Statistically significant with α=0.05; ** MENA: Middle East/North Africa.

**Table 4:** Regression analysis of mental distress and significant variables for young men.

Independent Variable	Coefficient	Standard Deviation	*t*-Value
Summer	−1.228	0.695	−1.766
Asia	0.648	0.357	1.815
Exercise (Moderate)	−0.316	0.206	−1.535
Exercise (High)	−0.636	0.217	−2.936 *
Dairy (Moderate)	−0.529	0.238	−2.226 *
Dairy (High)	−0.323	0.236	−1.367
Caffeine (Moderate)	0.397	0.228	1.744
Caffeine (High)	0.642	0.205	3.135 *
Meat (Moderate)	−0.679	0.335	−2.023 *
Meat (High)	−0.703	0.319	−2.201 *
Vegetables (Moderate)	0.081	0.220	0.368
Vegetables (High)	−0.146	0.245	−0.596
Beans (Moderate)	0.263	0.201	1.308
Beans (High)	0.248	0.307	0.811
Fish (Moderate)	0.438	0.231	1.900
Fish (High)	0.526	0.428	1.228
Fast Food (Moderate)	0.434	0.189	2.285 *
Fast Food (High)	0.988	0.248	3.983 *

* Statistically significant with α = 0.05.

**Table 5:** Regression analysis of mental distress and significant variables for mature men.

Independent Variable	Coefficient	Standard Deviation	*t*-Value
Asia	1.171	0.697	1.679
Middle East/North Africa (MENA)	1.382	0.415	3.330 *
Education	−0.536	0.246	−2.180 *
Exercise (Moderate)	−0.463	0.428	−1.080
Exercise (High)	−0.584	0.476	−1.228
Whole Grain (Moderate)	−0.703	0.423	−1.660
Whole Grain (High)	0.268	0.475	0.563
Dairy (Moderate)	0.391	0.448	0.872
Dairy (High)	−0.697	0.495	−1.409
Caffeine (Moderate)	−0.833	0.529	−1.575
Caffeine (High)	0.185	0.430	0.429
Fruits (Moderate)	−0.140	0.485	−0.288
Fruits (High)	0.529	0.492	1.075
Nuts (Moderate)	−0.918	0.428	−2.146 *
Nuts (High)	−1.069	0.570	−1.876
HGI Food (Moderate)	0.683	0.439	1.556
HGI Food (High)	0.211	0.503	0.418
Meat (Moderate)	0.262	0.599	0.437
Meat (High)	0.773	0.575	1.344
Vegetables (Moderate)	−0.690	0.450	−1.534
Vegetables (High)	−0.920	0.488	−1.885

* Statistically significant with α = 0.05.

**Table 6:** Summary results for the four different groups.

Description	Young Women	Mature Women	Young Men	Mature Men
Positive Associations	Exercice (M ^a^) Exercice (H ^b^) Breakfast (H)	Exercise (H) Breakfast (M) Breakfast (H) Fruits (H)	Exercise (H) Dairy (M) Meat (M) Meat (H)	Education Nuts (M)
Negative Associations	Spring Caffeine (H) Fast Food (M) Fast Food (H)	Spring Caffeine (H) Asia MENA *	Caffeine (H) Fast Food (M) Fast Food (H)	MENA *
Average K-6 Score ± Sd (95% CI)	8.1 ± 5.1 (7.80, 8.34)	5.7 ± 4.4 (5.36, 6.04)	7.5 ± 4.9 (7.12, 7.88)	5.4 ± 3.9 (4.87, 5.93)
Total Number	1147	628	641	207

^a^ M: Moderate, ^b^ H: High. * MENA: Middle East/North Africa; K-6: Kessler-6; Sd: Standard Deviation; CI: Confidence Interval.
